# Plant health evaluations of *Belonolaimus longicaudatus* and *Meloidogyne incognita* colonized bermudagrass using remote sensing

**DOI:** 10.21307/jofnem-2020-109

**Published:** 2020-11-06

**Authors:** Will L. Groover, Kathy S. Lawrence

**Affiliations:** Auburn University, 209 Rouse Life Science Building, Auburn, AL, 36849

**Keywords:** Abamectin, Fluensulfone, Fluopyram, Furfural, NDRE, NDVI

## Abstract

The objective of this study was to evaluate the ability of an unmanned aerial system (UAS) equipped with a multispectral sensor to track plant health in the presence of plant-parasitic nematodes in conjunction with nematicide applications. Four nematicides were evaluated for their ability to suppress *Belonolaimus longicaudatus* and *Meloidogyne incognita* in microplots, and three nematicides were evaluated on a golf course for their ability to suppress multiple plant-parasitic nematode genera. Visual ratings, Normalized Difference Vegetation Index (NDVI), and Normalized Difference RedEdge Index (NDRE) were reported throughout the trial to assess plant health. *B. longicaudatus* and *M. incognita* population density was significantly lowered by nematicide treatments in microplots and correlated with visual ratings, NDVI, and NDRE plant health ratings. On the golf course, all nematicides reduced total plant-parasitic nematode population density at 28, 56, and 84 days after treatment (DAT). Visual turf quality ratings, NDVI, and NDRE were positively correlated with lower nematode population density in the majority of evaluation dates. In the microplot and golf course settings, the parameters evaluated for plant health were correlated with plant-parasitic nematode population density: visual ratings, NDVI, and NDRE improved as nematode population density declined. These results show that remote sensing has the potential to be a beneficial tool for assessing plant-parasitic nematode infected bermudagrass.

Bermudagrass (*Cynodon* spp.) is one of the most commonly grown turfgrass species in the southern United States, and is very susceptible to a wide range of plant-parasitic nematodes (Crow, 2005). Examples of genera known to parasitize turfgrass in the United States include *Belonolaimus longicaudatus*, Rau (sting nematode), *Criconemoides* spp. (ring nematode), *Helicotylenchus* spp. (spiral nematode), *Hoplolaimus* spp., Cobb (lance nematode), and *Meloidogyne* spp. (root-knot nematode) (Sikora et al., 2001; Crow, 2005; Zeng et al., 2012). Feeding on the turfgrass root system by these nematodes results in inhibition of root growth, and can lead to a decrease in water and nutrient uptake (Lucas, 1982; White and Dickens, 1984; Trenholm and Unruh, 2005). This impact on root development, in turn, can lead to visible foliar symptoms including chlorosis and necrosis, wilting, and a thinning of the turf canopy (Johnson, 1970; Crow and Han, 2005; Aryal et al., 2017). With a wide range of potential plant-parasitic nematodes that can inhibit turfgrass growth, early and accurate detection of symptoms caused by nematode feeding could be important for more timely and effective management.

Over the past decade, there has been a significant rise in interest of unmanned aerial system (UAS) and remote multispectral sensing technology for turfgrass management. Spectral reflectance of the turfgrass canopy has been shown to provide valuable information on turfgrass species quality (Fitz-Rodriguez and Choi, 2002; Bremer et al., 2011), drought stress (Jiang and Carrow, 2007), nutrient levels (Volterrani et al., 2005; Caturegli et al., 2016), and fungal diseases (Sykes et al., 2020). A majority of this research has focused on using the normalized difference vegetation index [NDVI = (NIR−Red)/(NIR + Red), NIR = reflectance in the near-infrared region and Red = reflectance in the red light region], which is a commonly used plant stress indicator (Labus et al., 2002; Barton, 2012). Multiple research groups have shown that NDVI strongly correlates with a range of parameters related to turfgrass, including visual ratings, nitrogen applications, and shoot density (Trenholm et al., 1999; Bell et al., 2009; Caturegli et al., 2016). Trenholm et al. (2005) also reported significant improvement in NDVI by nematicides on turfgrass infested with *B. longicaudatus* in greenhouse evaluations. Thus, this is a proven vegetative index for rating bermudagrass in conjunction with visual ratings.

Another vegetation index used in crop stress management is the normalized difference red edge index [NDRE = (NIR−Red Edge)/(NIR + Red Edge), NIR = reflectance in the near-infrared region and Red Edge = reflectance in the change from red light to near-infrared light, approximately 680–750 nanometers] (Fitz-Rodriguez and Choi, 2002). NDRE has been shown to correlate with wheat and corn growth, and is a valuable tool for vegetative chlorophyll status (Horler et al., 1983). Krienke et al. (2017) found that NDRE values strongly correlate with nitrogen variability in maize production, and Tilling et al. (2007) found that NDRE was able to account for nitrogen level variability in wheat. In turfgrass, Hong et al. (2019) found that NDRE can be useful for drought stress analysis on creeping bentgrass (*Agrostis stolonifera* L.). Previous studies have also shown that NDRE has a positive correlation with clipping weight from hybrid bermudagrass (Fitz-Rodriguez and Choi, 2002).

While multiple studies have demonstrated the benefits of using these indices in turfgrass research, very few have evaluated the ability of these indices in turfgrass for their ability to track changes in plant health as a result of plant-parasitic nematodes. Research on *Heterodera glycines* (soybean cyst nematode, Ichinohe) has shown differences in spectral reflectance correlate with pathogen severity (Nutter et al., 2002; Bajwa et al., 2017). Joalland et al. (2018) were also able to correlate multiple spectral indices with yields of tolerant and susceptible sugar beets to *Heterodera schachtii* (beet cyst nematode, Schmidt). A research group in Brazil recently reported that red, red edge, and near-infrared spectral ranges were significant for discrimination of healthy coffee plants and coffee plants infected with *Meloidogyne* spp. at or above damage thresholds at an accuracy rate of 78% (Martins et al., 2017).

Visual ratings are the predominant means of assessing the impact of plant-parasitic nematode damage on turfgrass, yet visual evaluations and rating scales can lead to inconsistencies between evaluators, and assessing detailed turfgrass features can be subjective (Horst et al., 1984; Trenholm et al., 1999). Thus, the addition of NDVI and NDRE values in conjunction with visual turfgrass ratings may provide some stability in plant-parasitic nematode damage assessment. With this information in mind, the goal of this study was to evaluate UAS equipped with multispectral sensors for their ability to track plant health in the presence of plant-parasitic nematodes in conjunction with nematicide applications. Specifically, the objectives were to (i) assess turfgrass chemical nematicides for their ability to impact visual quality ratings, NDVI, and NDRE values on *M. incognita* and *B. longicaudatus* infected bermudagrass in microplots, and (ii) to advance to a golf course infested with multiple genera of plant-parasitic nematodes and assess the chemical nematicides ability to influence these same vigor ratings.

## Materials and methods

### Microplot establishment

Microplot trials were conducted at the Plant Science Research Center (PSRC) at Auburn University, AL during the summers of 2018 and 2019. Two trials were conducted: one to evaluate four nematicides for their ability to reduce the population density of *B. longicaudatus* population density, and the other to reduce the population density of *M. incognita*. Trials were established in 26.5-liter 35.5 cm diameter by 35.5 cm depth plastic tree pots nested one on top of the other with a brick in between to limit root growth by air pruning and filled with 100% sand. The microplots were submerged 30 cm in the soil with 5.5 cm of the pot above the soil line. Experiments were arranged in a randomized complete block design (RCBD) with five replications for each treatment. In 2018, ‘Tifway’ hybrid bermudagrass was laid as sod in each microplot and given ten weeks for establishment.

### Microplot nematode inocula

At the end of the 10-week period, *M. incognita* eggs were inoculated on half of the microplots at a rate of 50,000 eggs per pot on a weekly basis for 4 weeks to build up the nematode population density. The remaining microplots received an inoculation rate of 100 *B. longicaudatus* nematodes per pot on a weekly basis for 4 weeks. After the 4-week inoculation period, a 100-cm^3^ sample was taken from each plot to confirm *M. incognita* or *B. longicaudatus* population density. Inocula of *M. incognita* race 3 were originally isolated from an infested field at Plant Breeding Unit (PBU) at E.V. Smith Research Center of Auburn University in 2017 (Groover et al., 2019). Nematode eggs were extracted following a modified version of the methodology of Hussey and Barker (1973). The root mass was placed in a 0.625% sodium hypochlorite (NaOCl) solution and shaken for 4 min at 1 g-force on a Barnstead Lab Line Max Q 5000 E Class shaker (Conquer Scientific, San Diego, CA) and the nematode eggs were extracted by centrifugal floatation method (Jenkins, 1964). The contents were centrifuged at 427 g-forces for 1 min in a 1.14 specific gravity sucrose solution based on Jenkins (1964) methodology.

For the sting microplot experiment, *B. longicaudatus*, originally isolated from a golf course in east Alabama, was used as inoculum. The nematode population was obtained from total soil collected on a 25-µm pore sieve and the modified sucrose centrifugal flotation technique (Jenkins, 1964) was used to extract nematodes.

### Microplot nematicide treatments

Four nematicides were evaluated in the microplot setting during the study. Nematicides used were: (i) abamectin (Divanem; Syngenta, Greensboro, NC) at 0.89 L/ha; (ii) fluopyram (Indemnify; Bayer Environmental Science, Cary, NC) at 1.25 L/ha; (iii) fluensulfone (Nimitz Pro G; Control Solutions International, Pasadena, TX) at 134 kg/ha; and (iv) furfural (Multiguard Protect; Agriguard Company, LLC, Cranford, NJ) at 75 L/ha. An untreated treatment was included as the control. Abamectin, fluopyram, fluensulfone, and furfural were applied once at the start of the trial via a handheld spray bottle, and each treatment was diluted so that 10 mL of sprayed solution was the desired application rate per microplot. Fluensulfone was broadcast with a Scotts Easy Hand-Held Broadcast Spreader. All nematicides were watered with 0.64 cm of water after application.

### Microplot data collection

Data collection occurred at 0, 30, and 60 days after treatment (DAT) for both 2018 and 2019 trials. Data collected included nematode population density, visual turf quality assessment, NDVI, and NDRE. At each collection date, a 100-cm^3^ soil sample was taken from each plot. For *M. incognita,* population density, the soil sample was placed in a modified Baermann funnel (Castillo et al., 2013), and after 48 hr, juveniles were collected on a 25-µm pore sieve. For *B. longicaudatus*, the 100-cm^3^ sample was collected and nematodes were extracted using the modified centrifugal flotation technique as previously described. Once extracted, both *M. incognita* and *B. longicaudatus* presence were confirmed and quantified via a Nikon TSX 100 inverted microscope at 40-x magnification.

Turfgrass vigor was calculated using the National Turfgrass Evaluation Program (NTEP) guidelines (Parsons et al., 2015). Visual ratings consisted of a 1-9 rating scale, where 1 was very poor quality turf, 6 was minimal acceptable turf quality, and 9 was exceptional turf quality (Morris, 2004). Multispectral data were collected the same day that visual ratings were made. Drone flights occurred within 2 hr of solar noon on clear sunny days or light overcast days when ambient light was not changing. Specific dates of flights in 2018 were July 5, August 4, and September 3. Flight dates in 2019 were July 19, August 18, and September 17. A DJI Phantom 4 Professional drone (SZ DJI Technology Co.; Shenzhen, China) equipped with a MicaSense RedEdge-M (MicaSense, Inc.; Seattle, WA) was used for flight and image acquisition. Flight speed was 10 meters per second at a height of 35 meters, and imagery was spaced with 80% front overlap and 88% side overlap. The sensor measured wavelengths of blue (475 nm), green (560 nm), red (668 nm), red edge (RE, 717 nm), and near infrared (NIR, 840 nm). Automated image processing was performed with Pix4Dmapper (Pix 4D; Prilly, Switzerland), and wavelength values were calculated using ArcMap (Esri; Redlands, California). Wavelengths red, red edge, and near infrared were used to calculate NDVI and NDRE values for each plot in ArcMap. NDVI and NDRE were calculated as previously described.

### Field evaluations

A field trial was conducted in the summer of 2019 at Montevallo Golf Club in Montevallo, AL to assess the ability of multispectral imagery to track nematicidal responses on a plant-parasitic nematode infested golf course. Four putting greens consisting of ‘TifEagle’ hybrid bermudagrass were selected for the experiment: three with a history of high plant-parasitic nematode population density, and one with low population density. Each green was divided into four quadrants of approximately 75 m^2^, with three quadrants receiving a nematicide application, and one left as an untreated control. Treatments include: (i) untreated control; (ii) abamectin at 0.89 L/ha; (iii) fluopyram at 1.25 L/ha; and (iv) fluensulfone at 134 kg/ha.

### Field data collection

The trial was initiated on July 22. Nematode samples were collected at 0 DAT on July 22, at 28 DAT on August 19, at 56 DAT on September 16, and 84 DAT on October 14. At each sample date, six soil cores (2.5 cm × 10 cm) were taken at roughly equal intervals in a zigzag pattern across each quadrant of a green. Collected soil samples were mixed and a 100-cm^3^ subsample was processed to determine the plant-parasitic nematode population density from each quadrant. Nematodes were extracted by gravity sieving followed by sucrose centrifugation following the methodology of Jenkins (1964) as previously described. Plant-parasitic nematodes were confirmed and enumerated by a Nikon TSX 100 inverted microscope at 40-x magnification, and morphologically identified to genus or species if possible based upon ‘Pictorial Key to Genera of Plant-Parasitic Nematodes, 4th Edition’ (Mai and Lyon, 1975) and ‘Identification Guides for Most Common Genera of Plant-Parasitic Nematodes’ (Eisenback, 2002).

Drone flights occurred during the trial on approximately 14-day intervals. Flights occurred under full sun as close to solar noon as possible to limit environmental impact. Specific dates were July 22, August 5, August 19, September 3, September 16, September 30, and October 14. Flights were performed similarly to microplot evaluations, capturing NDVI, and NDRE values for each treatment quadrant. Visual turfgrass quality ratings were also conducted on the 1 to 9 scale using the NTEP guidelines.

### Statistical analysis

Data collected from microplot and field evaluations were statistically analyzed using SAS (version 9.4; SAS Institute, Cary, NC). Means separations were determined by Tukey’s multiple range test for each evaluation date (*P* ≤ 0.05). Rating methods (NTEP, NDVI, and NDRE) were compared statistically by the Pearson correlation coefficient at *P* ≤ 0.05, *P* ≤ 0.01, and *P* ≤ 0.001 to determine the strength of the linear relationship between nematode populations and foliar ratings.

## Results

### Microplot evaluations

**Figure 1:**
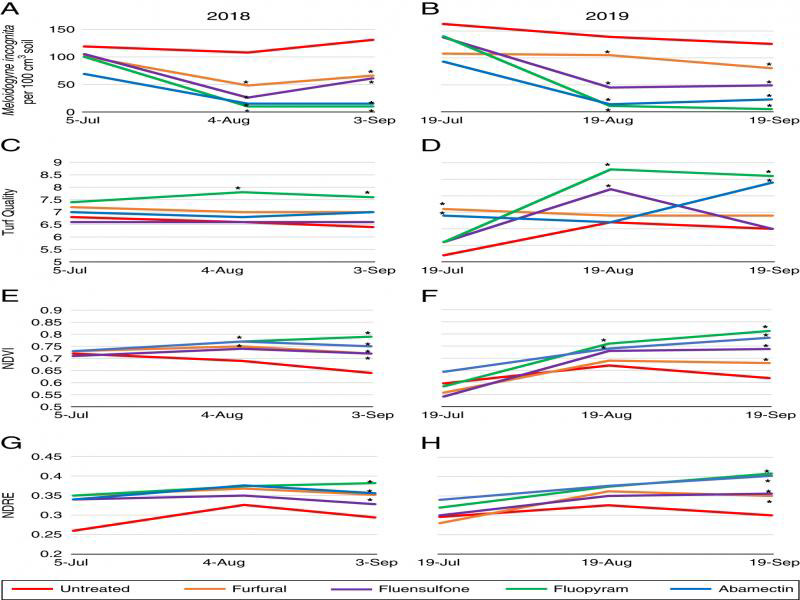
Auburn University *Meloidogyne incognita* nematicide microplot trials for 2018 and 2019 showing *M. incognita* population density for 2018 (A) and 2019 (B), visual turf quality ratings for 2018 (C) and 2019 (D), NDVI ratings for 2018 (E) and 2019 (F), and NDRE values for 2018 (G) and 2019 (H). *Different from the untreated control according to the pairwise comparison of each treatment to the untreated (Tukey’s, *P* ≤ 0.05).

*M. incognita* population density was significantly impacted by nematicide treatments in both 2018 and 2019 (Table 1). All nematicides significantly lowered *M. incognita* population density in August and September (30 and 60 days after treatment; DAT) for both years (*P* ≤ 0.05) (Fig. 1A, B). In 2018, only fluopyram significantly increased turf quality compared to the untreated control in August and September (*P* ≤ 0.05) (Fig. 1C). In 2019, both fluopyram and fluensulfone significantly increased turf quality compared to the untreated control in August, and fluopyram and abamectin significantly increased turf quality compared to the untreated control in September (*P* ≤ 0.05) (Fig. 1D). NDVI was significantly higher in both years for bermudagrass treated with fluopyram and abamectin in August, and all nematicides in September (*P* ≤ 0.05) (Fig. 1E, F). No significant differences were observed for NDRE in August 2018 and fluopyram, abamectin, and furfural had a significantly higher NDRE value compared to the untreated plots in September (*P* ≤ 0.05) (Fig. 1G). In 2019, all nematicides significantly increased NDRE in September compared to the untreated control (*P* ≤ 0.05) (Fig. 1H).

**Table 1.** Pearson correlation coefficients^†^ resulting from linear correlation of data parameters from 2018 and 2019 *Meloidogyne incognita* infested bermudagrass microplots in Auburn, AL.

	2018			2019		
	July	August	September	July	August	September
*Turf visual quality*^*a*^						
NDVI^b^	0.59**	NS	NS	NS	NS	0.63***
NDRE^c^	NS	NS	NS	NS	NS	0.66***
*NDRE*						
NDVI	NS	0.72***	0.93***	0.60***	0.72***	0.95***
*Meloidogyne incognita*						
Turf visual quality	NS	−0.39*	NS	−0.64*	−0.39*	−0.59**
NDVI	NS	−0.48*	−0.76***	NS	−0.37*	−0.82***
NDRE	NS	NS	−0.61**	NS	NS	−0.76***

**Notes:** ^†^NS, *,**,***Tests of linear correlation between variables were not significant (NS) or were significant at *P* ≤ 0.05, *P* ≤ 0.01, or *P* ≤ 0.001, respectively. ^a^Turf visual quality ratings were assigned on a 1 to 9 scale, where 1 = poorest turf quality, 6 = minimally acceptable turf quality, and 9 = exceptional turf quality; ^b^NDVI (Normalized Difference Vegetation Index) collected using a MicaSense Rededge-M (MicaSense, Inc.; Seattle, WA) sensor; ^c^NDRE (Normalized Difference RedEdge Index) collected using a MicaSense Rededge-M (MicaSense, Inc.; Seattle, WA) sensor.

**Figure 2:**
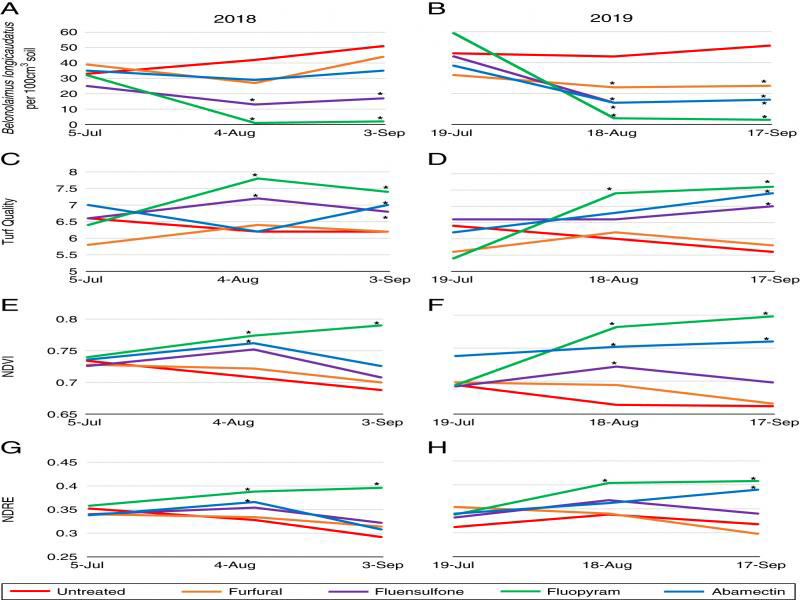
Auburn University *Belonolaimus longicaudatus* nematicide microplot trials for 2018 and 2019 showing *B. longicaudatus* population density for 2018 (A) and 2019 (B), visual turf quality ratings for 2018 (C) and 2019 (D), NDVI ratings for 2018 (E) and 2019 (F), and NDRE values for 2018 (G) and 2019 (H). *Different from the untreated control according to the pairwise comparison of each treatment to the untreated (Tukey’s, *P* ≤ 0.05).

In 2018, fluopyram and fluensulfone significantly reduced *B. longicaudatus* population density compared to the untreated control in August and September, and in 2019 fluopyram, fluensulfone, and furfural significantly reduced population density in August and September compared to the untreated control (*P* ≤ 0.05) (Fig. 2A, B). Turf quality was significantly improved by fluopyram and fluensulfone compared to the untreated control in August and abamectin, fluopyram, and fluensulfone in September in 2018 (*P* ≤ 0.05) (Fig. 2C). In 2019, fluopyram significantly increased turf quality compared to the untreated control in August, and abamectin, fluopyram, and fluensulfone increased turf quality compared to the untreated control in September (*P* ≤ 0.05) (Fig. 2D). In 2018, abamectin and fluopyram significantly increased NDVI compared to the untreated control in August, and fluopyram significantly increased NDVI compared to the untreated control in September (*P* ≤ 0.05) (Fig. 2E). For 2019, NDVI was significantly improved by abamectin, fluensulfone, and fluopyram in August, and abamectin and fluopyram in September (*P* ≤ 0.05) (Fig. 2F). Abamectin and fluopyram both significantly improved NDRE in August of 2018 compared to the untreated control, and fluopyram significantly improved NDRE in September compared to the untreated control (*P* ≤ 0.05) (Fig. 2G). In 2019, only fluopyram significantly improved NDRE in August compared to the untreated, and fluopyram and abamectin significantly improved NDRE compared to the untreated control in September (*P* ≤ 0.05) (Fig. 2H).

Turfgrass visual ratings, NDVI, and NDRE were correlated with *M. incognita* and *B. longicaudatus* population density in both 2018 and 2019. In 2018, turf visual ratings significantly correlated with *M. incognita* population density in August (*P* ≤ 0.05), and all three sample dates in 2019 (*P* ≤ 0.05) (Table 1). NDVI was significantly correlated with *M. incognita* population density in August (*P* ≤ 0.05) and September (*P* ≤ 0.001) in 2018 and 2019. NDRE was significantly correlated with *M. incognita* population density in September in 2018 (*P* ≤ 0.01) and 2019 (*P* ≤ 0.001). *B. longicaudatus* population density was significantly correlated with turf visual ratings at July (*P* ≤ 0.001) and September dates (*P* ≤ 0.01) in 2018, and August and September dates (*P* ≤ 0.01) in 2019 (Table 2). Both NDVI and NDRE were significantly correlated with *B. longicaudatus* population density in September 2018 (*P* ≤ 0.001), and all sample dates in 2019 (*P* ≤ 0.01) (Table 2).

**Table 2.** Pearson correlation coefficients^†^ resulting from linear correlation of data parameters from 2018 and 2019 *Belanolaimus lonigcaudatus* infested bermudagrass microplots in Auburn, AL.

	2018			2019		
	July	August	September	July	August	September
*Turf visual quality*^*a*^						
NDVI^b^	NS	NS	0.63***	NS	0.43*	0.41*
NDRE^c^	NS	NS	0.66***	NS	0.45*	0.56**
*NDRE*						
NDVI	0.60**	0.72***	0.96***	0.91***	0.96***	0.84***
*Belonolaimus longicaudatus*						
Turf visual quality	−0.64***	NS	−0.56**	NS	−0.56**	−0.54**
NDVI	NS	NS	−0.57**	−0.51**	−0.57***	−0.56**
NDRE	NS	NS	−0.61**	−0.53**	−0.61***	−0.63**

**Notes:** ^†^NS, *,**,***Tests of linear correlation between variables were not significant (NS) or were significant at *P* ≤ 0.05, *P* ≤ 0.01, or *P* ≤ 0.001, respectively. ^a^Turf visual quality ratings were assigned on a 1 to 9 scale, where 1 = poorest turf quality, 6 = minimally acceptable turf quality, and 9 = exceptional turf quality; ^b^NDVI (Normalized Difference Vegetation Index) collected using a MicaSense Rededge-M (MicaSense, Inc.; Seattle, WA) sensor; ^c^NDRE (Normalized Difference RedEdge Index) collected using a MicaSense Rededge-M (MicaSense, Inc.; Seattle, WA) sensor.

Turfgrass visual ratings, NDVI, and NDRE were highly correlated with each other throughout both the *M. incognita* and *B. longicadatus* microplot evaluations. In the *M. incognita* trial, turf visual quality was positively correlated with NDVI in July (*P* ≤ 0.01) of 2018, and September of 2019 (*P* ≤ 0.001) (Table 1). NDRE was positively correlated with turf visual quality in September of 2019 (*P* ≤ 0.001). NDVI and NDRE were positively correlated with each other at every evaluation date in 2018 and 2019 (*P* ≤ 0.001), except for July in 2018 (Table 1). For the *B. longicaudatus* trial, turf visual quality was positively correlated with NDVI and NDRE in September of 2018 (*P* ≤ 0.001), and both August and September of 2019 (*P* ≤ 0.05) (Table 2). NDVI and NDRE were positively correlated to each other at every flight date in 2018 and 2019 (*P* ≤ 0.001).

### Field evaluations

**Figure 3:**
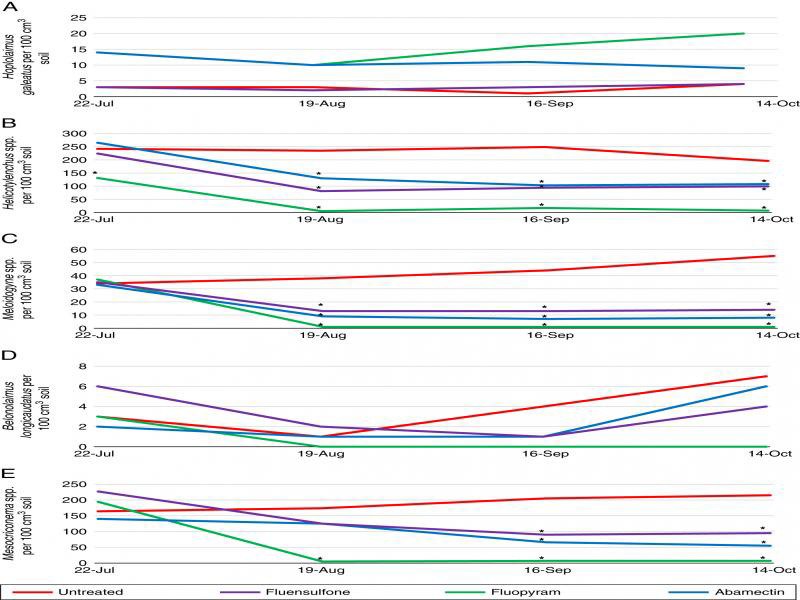
Plant-parasitic nematode genera identified at Montevallo Golf Course, Montevallo, AL in 2019. Genera include *Hoplolaimus* spp. (A), *Helicotylenchulus* spp. (B), *Meloidogyne* spp. (C), *Belonolaimus longicaudatus* (D), and *Criconemoides* spp. (E). *Different from the untreated control according to the pairwise comparison of each treatment to the untreated (Tukey’s, *P* ≤ 0.05).

**Figure 4:**
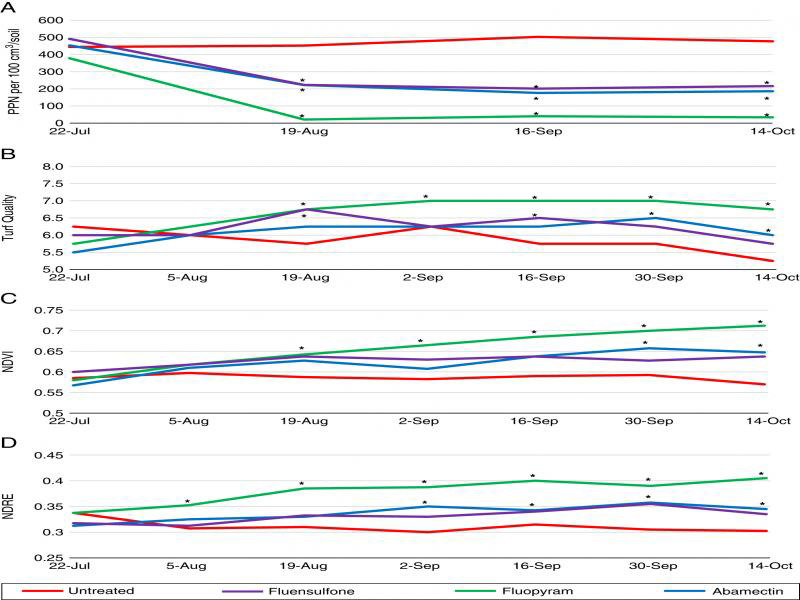
Nematicide effects on total plant-parasitic nematode population density (A), visual turf quality (B), NDVI (C), and NDRE (D) at Montevallo Golf Course, Montevallo AL, 2019. *Different from the untreated control according to the pairwise comparison of each treatment to the untreated (Tukey’s, *P* ≤ 0.05).

Five plant-parasitic nematode genera were identified throughout the duration of the 2019 field trial at Montevallo Golf Club. These include *Hoplolaimus* spp. (lance nematode, Fig. 3A), *Helicotylenchus* spp. (spiral nematode, Fig. 3B), *Meloidogyne* spp. (Fig. 3C), *B. longicaudatus* (Fig. 3D), and *Criconemoides* spp. (ring nematode, Fig. 3E). All nematicides reduced total plant-parasitic nematode population density on August 19, September 16, and October 14 compared to the untreated control (*P* ≤ 0.05) (Fig. 4A). Fluopyram significantly improved visual turf quality and NDVI on August 19, September 3, September 16, September 30, and October 14 (*P* ≤ 0.05) (Fig. 4B, C), and NDRE on August 5, August, 19, September 3, September 16, September 30, and October 14 (*P* ≤ 0.05) (Fig. 4D) compared to the untreated control. Abamectin and fluopyram also significantly improved visual turf quality at two evaluation dates apiece (*P* ≤ 0.05) (Fig. 4B). Abamectin significantly improved NDVI September 30 and October 14 compared to the untreated control, and significantly improved NDRE on September 3, September 30, and October 14 (*P* ≤ 0.05) (Fig. 4C, D). Fluensulfone significantly improved NDRE on September 30 (*P* ≤ 0.05) (Fig. 4D).

Total plant-parasitic nematode population density was highly correlated with turfgrass visual ratings, NDVI and NDRE. Correlation occurred with visual ratings on July 22 (*P* ≤ 0.01), August 19, September 16, and October 14 (*P* ≤ 0.001) (Table 3). Nematode population density and NDVI were correlated on July 22 (*P* ≤ 0.05), August 19, September 16, and October 14 (*P* ≤ 0.001) (Table 3). NDRE and nematode population density were correlated on August 19 (*P* ≤ 0.05), September 16 (*P* ≤ 0.01), and October 14 (*P* ≤ 0.001) (Table 3). NDVI and turf quality ratings were positively correlated at five of the seven evaluation dates, and NDRE and turf quality were positively correlated at four of the seven evaluation dates (*P* ≤ 0.01) (Table 3). NDVI and NDRE were positively correlated at five of the seven evaluation dates (*P* ≤ 0.05) (Table 3).

**Table 3.** Pearson correlation coefficients^†^ resulting from linear correlation of vigor ratings and plant-parasitic nematode density from four bermudagrass putting greens treated with three different nematicides in 2019 in Montevallo, AL.

	July 22	August 5	August 19	September 3	September 16	September 30	October 14
*Turf visual quality*^*a*^							
NDVI^b^	NS	NS	0.63**	0.70**	0.62**	0.78***	0.80***
NDRE^c^	NS	NS	NS	0.59*	0.51*	0.67**	0.60**
*NDVI*							
NDRE	NS	NS	0.49*	0.76***	0.93***	0.79***	0.68**
*Total plant-parasitic nematodes*							
Turf visual quality	−0.64**		−0.81***		−0.85***		−0.84***
NDVI	−0.47*		−0.84***		−0.80***		−0.82***
NDRE	NS		−0.59*		−0.70**		−0.70**

**Notes:** ^†^NS, *,**,***Tests of linear correlation between variables were not significant (NS) or were significant at *P* ≤ 0.05, *P* ≤ 0.01, or *P* ≤ 0.001, respectively. ^a^Turf visual quality ratings were assigned on a 1 to 9 scale, where 1 = poorest turf quality, 6 = minimally acceptable turf quality, and 9 = exceptional turf quality; ^b^NDVI (Normalized Difference Vegetation Index) collected using a MicaSense Rededge-M (MicaSense, Inc.; Seattle, WA) sensor; ^c^NDRE (Normalized Difference RedEdge Index) collected using a MicaSense Rededge-M (MicaSense, Inc.; Seattle, WA) sensor.

## Discussion

Evaluating remote sensing technology for its ability to improve turfgrass management is very important, and the potential uses for UAS in the turfgrass industry are widespread. This study confirmed previous reports of the correlation between both NDVI and NDRE and turfgrass quality (Bell et al., 2009; Caturegli et al., 2016; Hong et al., 2019). These results also indicate that NDVI and NDRE captured via remote sensing are reliable metrics for incorporating into evaluating turfgrass damage caused by plant-parasitic nematodes. In all nematicide trials, a positive correlation was observed for multiple evaluation dates between visual turfgrass quality and both NDVI and NDRE, meaning that as turfgrass visually improved, so did NDVI and NDRE. Conversely, multiple evaluation dates for all trials displayed a negative correlation between plant-parasitic nematode genera and both NDVI and NDRE. As plant-parasitic nematode population density increased, both NDVI and NDRE declined.

*M. incognita* and *B. longicaudatus* population density were high in microplot trials for both 2018 and 2019, with initial population density at or near levels that have previously been reported to recommend a nematicide application in Alabama (Sikora et al., 2001). For the *M. incognita* infested microplots, the strongest correlations between nematode population density and NDVI and NDRE occurred in September for both years. In fact, nematode population density had a stronger correlation with both NDVI and NDRE in 2018 and 2019 in September compared to visual turf quality ratings. Similar results were observed in the *B. longicaudatus* microplots, with strongest correlations of visual turfgrass quality, NDVI, and NDRE compared to population density occurring in September. While both NDVI and NDRE had a higher correlation with population density in September numerically compared to the visual ratings, only 2018 correlations were higher. This is similar to greenhouse evaluations by Trenholm et al. (2005), who found significant improvement of NDVI in *B. longicaudatus* infested turfgrass by nematicide treatment. Previous research has also shown that each of the nematicides evaluated in this study can be effective tools for lowering plant-parasitic nematode population density (Blackburn et al., 1996; Aryal et al., 2017; Baird et al., 2017; Crow *et al.*, 2017). Feeding by plant-parasitic nematodes inhibit root growth and function, so as root growth and development improve after a nematicide treatment, turfgrass vigor should improve as well (Crow, 2005; Luc et al., 2007).

Another interesting finding from the microplot evaluations was that NDVI and NDRE ratings indicated nematicide reductions of nematode population density more frequently than the visual turfgrass ratings. For example, in the 2018 *M. incognita* microplot trial, only fluopyram improved visual turfgrass quality compared to the untreated control in August and September. However, two of the four nematicides had an improvement in NDVI in August compared to the untreated control, and all nematicides improved NDVI in September compared to the untreated control. Three of the four nematicide treatments also improved NDRE compared to the untreated control in September. A similar trend was observed in 2019 *M. incognita* inoculated microplots, as more nematicide treatments reduced nematode numbers and increased NDVI and NDRE compared to the untreated control than with visual turfgrass ratings.

While NDVI, NDRE, and turf quality were often found to be significantly correlated with plant-parasitic nematode population density, it is worth noting that several of these values were numerically low. Across visual turf quality, NDVI, and NDRE, the *B. longicaudatus* microplot trials had more evaluation dates with a significant linear correlation with plant-parasitic nematode population density compared to the *M. incognita* microplot trials. Even though some of the correlation coefficients in both *B. longicaudatus* and *M. incognita* microplot trials were low, this indicates that there may be a stronger impact on turfgrass by *B. longicaudatus* than *M. incognita*. In a similar study comparing NDVI to turfgrass quality, Bremer et al. (2010, 2011) noted that differences were frequently observed in NDVI among turfgrasses even when all were visually rated at the same quality level. While the mechanism for this occurrence is not fully understood, they theorize that factors such as chlorophyll content, plant water status, and leaf cell constituents may be influencing red and NIR reflectance.

At the field site, multiple genera of plant-parasitic nematodes were identified. While average population density for each individual genus of nematode was not, on its own, above economic thresholds, it is clear that the combined presence of the total nematode population density did have a detrimental impact on the turfgrass. Field results from this study were consistent with microplot data. Visual turf quality and NDVI were both correlated with total plant-parasitic nematode population density at all evaluation dates, and NDRE was correlated with nematode population density at all but the first evaluation date.

Visual evaluations can be inconsistent among evaluators, and studies have shown that even the same turfgrass evaluator may not be consistent on a day-to-day basis (Horst et al., 1984; Bell et al., 2009). However, digital imagery captured and processed quickly for immediate use, can help eliminate potential inconsistencies in turfgrass evaluation. While it is still vital to use visual assessments in conjunction with proper soil sampling to diagnose plant-parasitic nematode damage on turfgrass, the results of this research show that UAS-assisted multispectral imagery analysis may provide an additional tool to help assess and track the impact of plant-parasitic nematodes on intensively maintained turfgrass.
